# Interferential current stimulation during dental facial treatment improves masticatory function

**DOI:** 10.1038/s41598-026-36307-6

**Published:** 2026-01-23

**Authors:** Masayuki Hara, Norimasa Hara, Yoshitaka Oku

**Affiliations:** 1 Hara Dental Clinic, 2203 Yokomichi, Nagakute, Aichi 480-1129 Japan; 2 EuSense Medical Co., Ltd., 3-12 Kasumi-Cho, Nishinomiya, Hyogo 662-0052 Japan; 3 Department of Neurophysiology, Hyogo Medical University, 1-1 Mukogawa-Cho, Nishinomiya, Hyogo 663-8501 Japan

**Keywords:** Transcutaneous interferential current stimulation, Orofacial neuromodulation, Masticatory performance, Dysphagia prevention, Occlusal force, Randomized crossover trial, Health care, Medical research, Neuroscience

## Abstract

Interferential current transcutaneous stimulation (IW) has been reported to influence swallowing-related sensorimotor function, although its immediate effects on mastication remain unclear. In a randomized, sham-controlled crossover study, we examined whether IW stimulation applied over the masseter region is associated with immediate improvements in masticatory performance. The primary endpoint was glucose elution from a standardized gummy jelly, an indicator of masticatory and occlusal function; the secondary endpoint was a 0–100 mm VAS of chewing ease. Mixed-effects modeling adjusted for period and sequence. IW produced immediate, within-session gains in occlusal function: glucose elution increased after IW (mean difference + 31.5 mg/dL, p < 0.001) but decreased with sham (− 19.8 mg/dL, p = 0.0019). VAS improved within both treatments, with a larger median gain under IW, and the mixed-effects model favored IW over sham. Although this study does not directly assess neural circuits, the observed pattern is consistent with peripheral sensory influences on masticatory output. Expectancy effects and other nonspecific contributors cannot be fully excluded and should be addressed in future studies using active control conditions and direct physiological measures. IW may offer a rapid, noninvasive adjunct to oral rehabilitation; confirmatory trials should incorporate direct force/EMG, jaw kinematics, and salivary measures, and assess durability and clinical outcomes.

Trial registration: UMIN-CTR UMIN000058249 (registered 2025-07-01). Direct link: https://center6.umin.ac.jp/cgi-open-bin/ctr/ctr_view.cgi?recptno=R000066586.

## Introduction

Dental facial treatment (DFT) is a clinical intervention in which an interferential wave (IW)-equipped electrical stimulation device is applied to the orofacial region during routine dental care. Beyond orofacial care, such stimulation may influence orofacial motor function through peripheral sensory input.

Transcutaneous electrical sensory stimulation to the neck using interferential current (IFCS) has been widely studied in the field of dysphagia rehabilitation. Experimental work in healthy subjects demonstrated that IFCS facilitates the swallowing reflex^1^, while clinical trials in patients with dysphagia have shown that IFCS shortens pharyngeal response time and improves swallowing efficiency^2^. Longer-term application has been reported to enhance airway defense mechanisms and increase oral caloric intake^3^, thereby contributing to improved nutritional status and clinical outcomes. Importantly, IFCS is considered safe, with no adverse effects on vital signs even in frail populations^4^. Collectively, these findings indicate that IFCS can rapidly and reproducibly influence swallowing-related function via peripheral sensory stimulation, supporting its clinical utility in dysphagia rehabilitation. Moreover, a recent study reported improvements in both masticatory and swallowing functions with IFCS^5^.

Building upon this evidence, we hypothesized that IW stimulation delivered during DFT may be associated with immediate improvements in masticatory performance. Clinically, chewing ability is not merely a biomechanical endpoint: difficulty in chewing and suboptimal oral conditions are linked to adverse outcomes, including an increased risk of aspiration pneumonia^6,7^. If DFT using IW stimulation can improve chewing performance, it would represent a simple, non-invasive method with potential applications in both dental practice and geriatric care. Ultimately, such an approach could contribute not only to oral health and quality of life but also to the prevention of dysphagia-related complications.

The present study was designed to test this hypothesis using objective and subjective measures of masticatory performance. We conducted a randomized crossover trial to compare masticatory performance with and without interferential current stimulation during DFT.

## Results

Forty-eight participants were enrolled. Two did not attend the second visit and were withdrawn—one due to eye surgery and one due to a family member’s emergency hospitalization—leaving 46 participants who completed both periods and were included in the crossover analyses. No adverse events were observed. The sample comprised 29 females and 17 males. Sex was recorded and reported descriptively; given the crossover design and limited sample size, the analysis plan did not include sex as a covariate, and no sex-stratified analyses were conducted.

### Primary outcome: glucose concentration

**Fig. 1:**
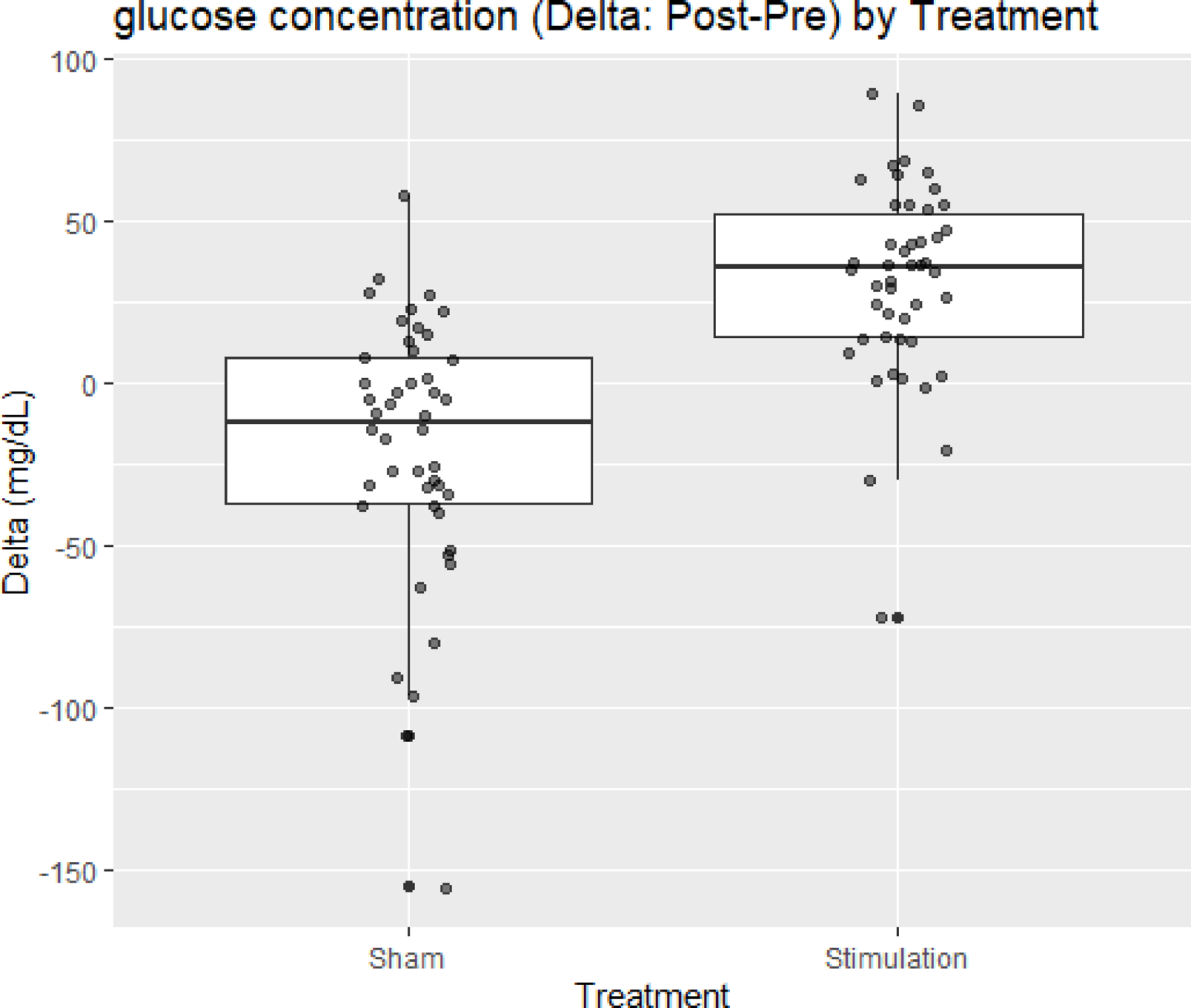
Δ glucose (Post − Pre) by treatment*.* Box-and-whisker plots display participant-level changes in glucose concentration (mg/dL) for no stimulation (**N**) and stimulation (**S**). Center line indicates the median; box, interquartile range (IQR); whiskers, 1.5 × IQR (points beyond whiskers are outliers, if present). Positive values denote increases from Pre to Post. A linear mixed-effects model (Δ ~ Treatment + Period + Sequence + (1|ID)) showed a significantly greater improvement under **S** versus **N** (estimate + 51.2 mg/dL, SE 7.5, df 88, *t* = 6.85, *p* < 0.001). *n* = 46 participants (92 observations).

Ninety-two observations from 46 participants were analyzed. In the primary mixed-effects model adjusting for Period and Sequence, Treatment was significantly associated with Δ glucose, with greater improvement under **S** (Stimulation) versus **N** (Sham, no stimulation) (estimate + 51.2 mg/dL, SE 7.5, 95% CI ≈ 36–66 mg/dL, df 88, *t* = 6.85, p < 0.001) (Table 1 and Fig. 1). Period (β =  − 5.17 mg/dL, SE 7.48, *p* = 0.49) and Sequence (β =  + 6.17 mg/dL, SE 7.48, *p* = 0.41) were not significant. The random-intercept variance was near zero (singular fit), indicating negligible between-subject heterogeneity relative to the residual variance; fixed-effect inferences are therefore essentially unchanged under a fixed-effects model. The estimated treatment effect (51.2 mg/dL) exceeded the prespecified minimal clinically important difference (MCID) of 30 mg/dL.

**Table 1:** Fixed effects from the glucose mixed-effects model (Δ as outcome).

Effect	Estimate (mg/dL)	Standard error (SE)	Degrees of freedom (df)	t value	p-value
Intercept*	−20.261	7.477	88	−2.710	0.0081
Treatment (S vs. N)	51.217	7.477	88	6.850	< 0.001
Period (2 vs. 1)	−5.174	7.477	88	−0.692	0.49
Sequence (B vs. A)	6.174	7.477	88	0.826	0.41

* Treatment N: sham (no stimulation), S: stimulation. Sequence = A: S → N, B: N → S.

* Reference: Treatment = N, Period = 1, Sequence = A.

* Intercept corresponds to the reference levels.

Carryover term was attempted but was not identifiable (rank-deficient); coefficient dropped automatically.

**Fig. 2:**
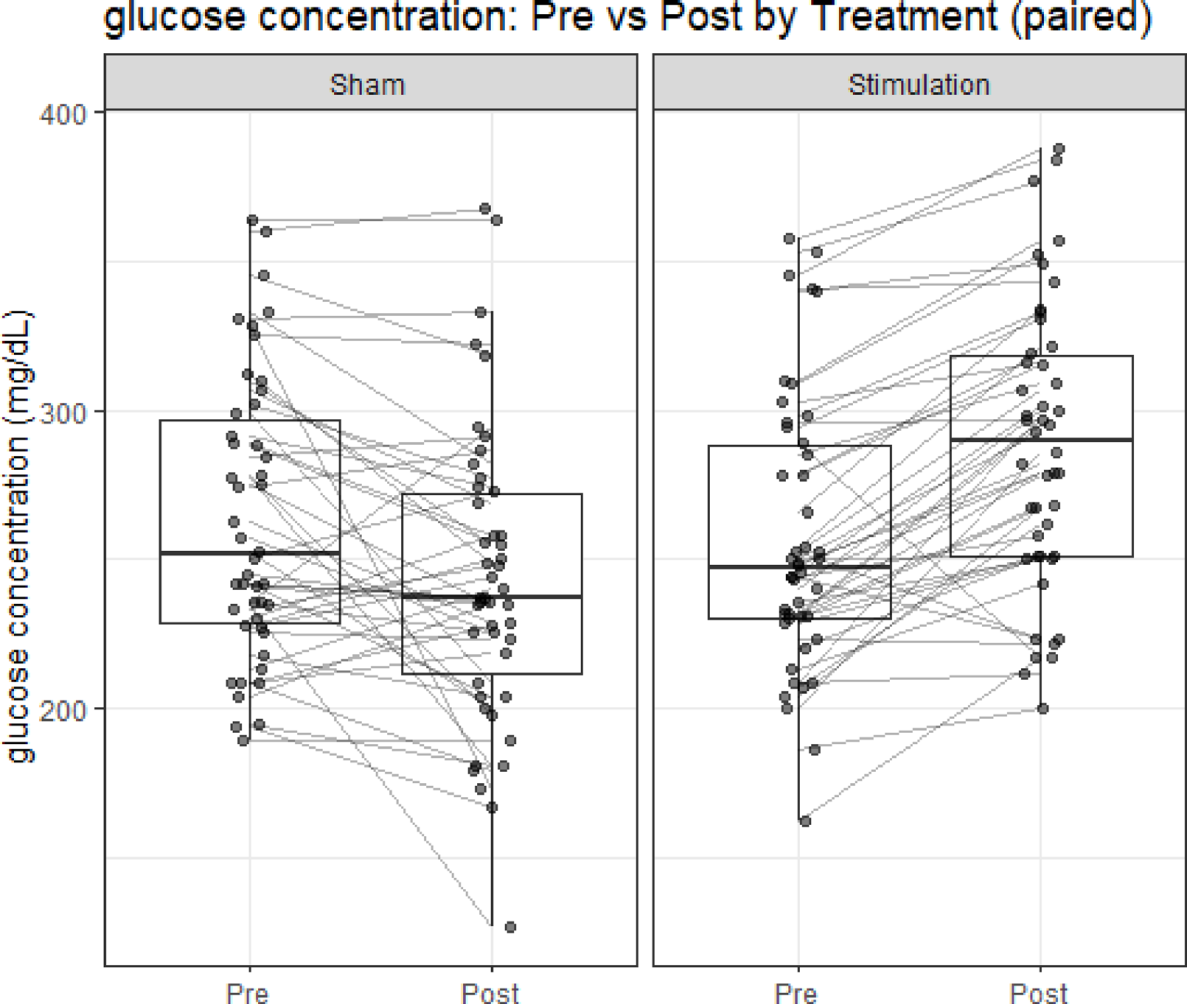
Within-treatment changes in glucose concentration (paired). Box-and-whisker plots show glucose concentration (mg/dL) at Pre and Post for each treatment (no stimulation (**N**) and stimulation (**S**)). Horizontal lines denote medians, boxes interquartile ranges, and whiskers 1.5 × IQR. Individual dots represent participants, with lines connecting paired Pre–Post measurements. Glucose decreased significantly in Treatment **N** (mean difference =  − 19.8 mg/dL, *p* = 0.0019) and increased significantly in Treatment **S** (mean difference =  + 31.5 mg/dL, *p* < 0.001). These within-treatment results are consistent with the primary crossover mixed-effects analysis, which demonstrated greater improvement under Treatment **S** than under Treatment **N**.

As shown in Fig. 2, for Treatment N, the mean glucose decreased from Pre 263 mg/dL to Post 243 mg/dL (mean difference [Post − Pre] =  − 19.8 mg/dL, 95% CI − 31.9 to − 7.7 mg/dL; *t*(45) =  − 3.29; *p* = 0.0019). For Treatment S, the mean glucose increased from Pre 257 mg/dL to Post 288 mg/dL (mean difference [Post − Pre] =  + 31.5 mg/dL, 95% CI 22.6 to 40.4 mg/dL; *t*(45) = 7.15; *p* < 0.001). Within-treatment paired *t*-tests were reported as descriptive; Holm adjustment across the two tests was also examined and did not change inference. These within-treatment findings are consistent with the primary mixed-effects analysis, showing a greater improvement under S than under N.

### Secondary outcome: VAS

**Fig. 3:**
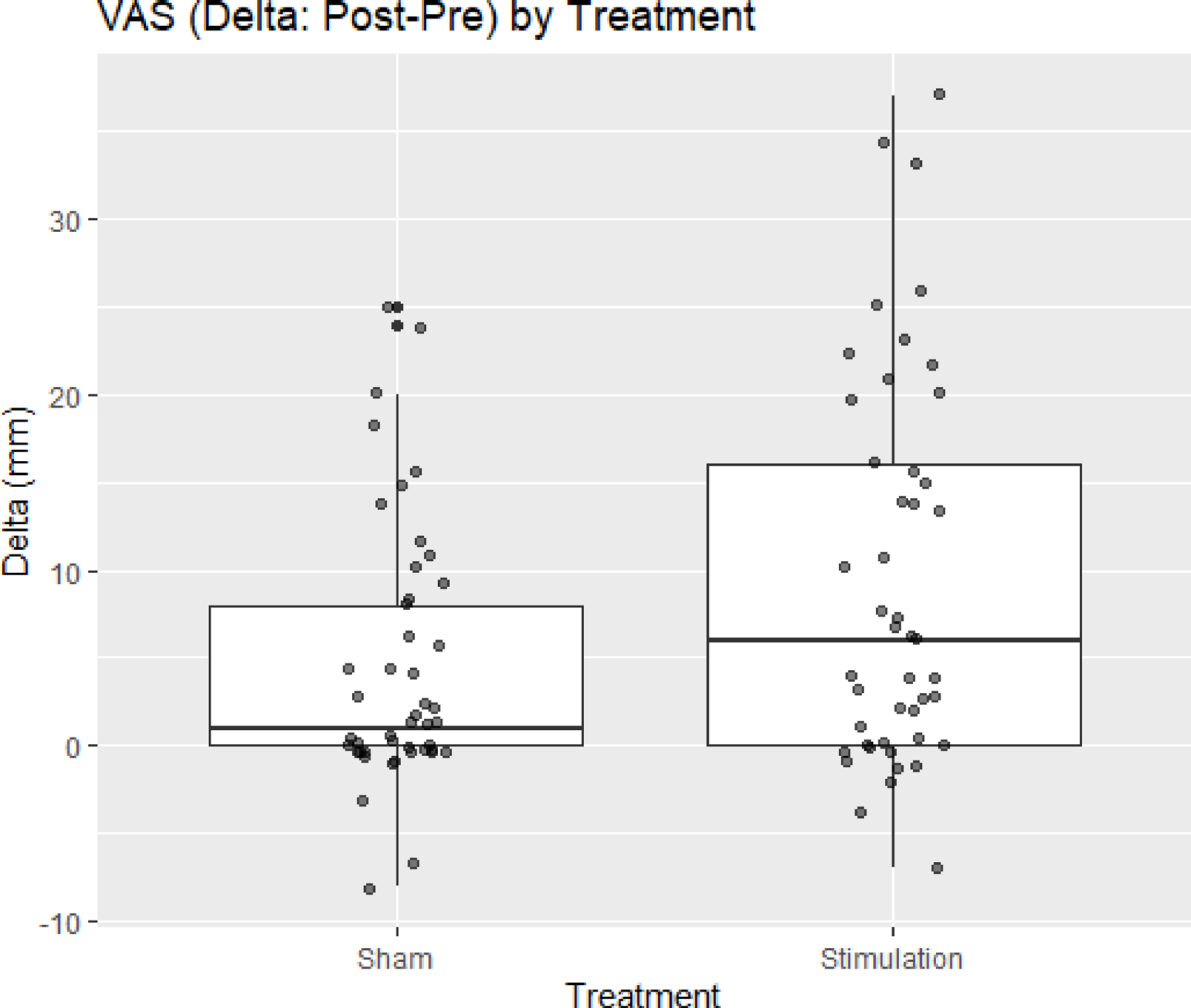
ΔVAS (Post − Pre) by treatment. Box-and-whisker plots show participant-level changes in VAS (mm) for no stimulation (**N**) and stimulation (**S**). Positive values indicate an increase (improvement) from Pre to Post. The center line denotes the median; the box, the interquartile range (IQR); and whiskers, 1.5 × IQR (points represent individual participants). A linear mixed-effects model (ΔVAS ~ Treatment + Period + Sequence + (1|ID)) demonstrated significantly greater improvement under **S** versus **N** (estimate + 5.00 mm, SE 1.61, df 44, *t* = 3.12, *p* = 0.003; approximate 95% CI 1.77–8.23 mm). *n* = 46 participants (92 observations).

In the VAS analysis (92 observations from 46 participants), Treatment was significantly associated with greater improvement, with **S** (Stimulation) exceeding **N** (Sham, no stimulation) by + 5.00 mm (SE 1.61, df 44, *t* = 3.12, *p* = 0.003; approximate 95% CI 1.77 to 8.23 mm) (Table 2 and Fig. 3). Period (β = + 1.65 mm, SE 1.61, *p* = 0.31) and Sequence (β =  + 1.74 mm, SE 2.27, *p* = 0.45) were not significant. The random-intercept variance (29.59) relative to the residual variance (59.28) yielded an intraclass correlation of roughly 0.33, indicating non-negligible within-subject correlation and supporting use of a mixed-effects approach.

**Table 2:** Fixed effects from the VAS mixed-effects model (Δ as outcome).

Effect	Estimate (mm)	Standard error (SE)	Degrees of freedom (df)	t value	p-value
Intercept*	2.783	1.966	79.216	1.416	0.16
Treatment (S vs. N)	5.000	1.605	44.000	3.115	0.003
Period (2 vs. 1)	1.652	1.605	44.000	1.029	0.31
Sequence (B vs. A)	1.739	2.269	44.000	0.766	0.45

* Treatment N: sham (no stimulation), S: stimulation. Sequence = A: S → N, B: N → S.

* Reference: Treatment = N, Period = 1, Sequence = A.

* Intercept corresponds to the reference levels.

**Fig. 4:**
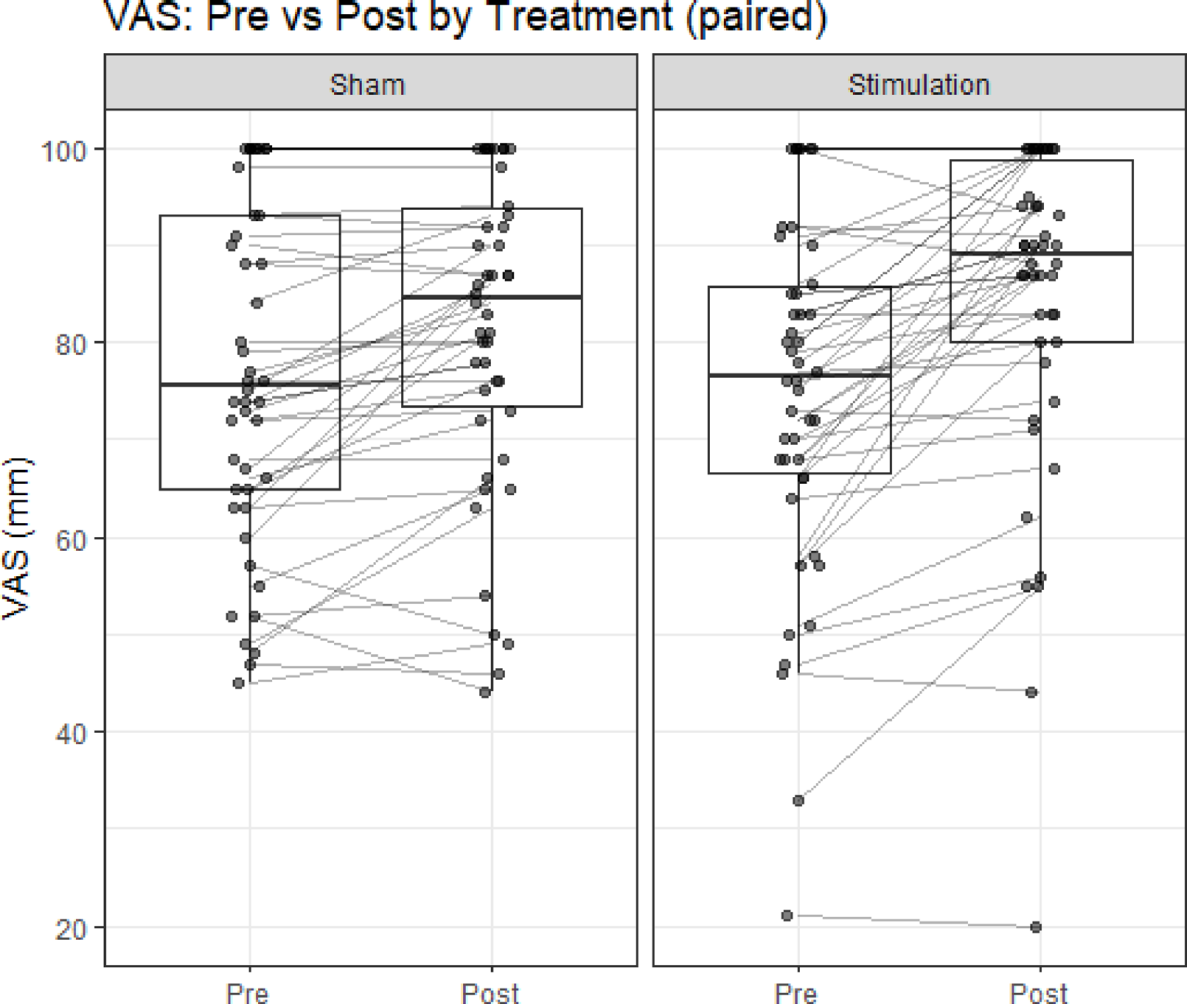
Within-treatment changes in VAS (paired). Box-and-whisker plots display VAS (mm) at Pre and Post for each treatment—no stimulation (**N**) and stimulation (**S**). The center line denotes the median; boxes indicate the interquartile range (IQR); whiskers extend to 1.5 × IQR; points represent individual participants, with lines connecting paired Pre–Post measurements. Within-treatment VAS increased significantly for both conditions by the paired Wilcoxon signed-rank test: **N** (Hodges–Lehmann Δ = 6.0 mm, 95% CI 2.5–9.5 mm), S (Δ = 10.5 mm, 95% CI 6.5–14.5 mm). These within-treatment results are consistent with the mixed-effects analysis indicating greater improvement under **S** than under **N**.

Normality of the paired pre–post VAS differences (Δ = Post − Pre) was rejected for both treatments by the Shapiro–Wilk test (N: W = 0.860, *p* < 0.001; S: W = 0.911, *p* = 0.0018). Accordingly, within-treatment changes were analyzed using the paired Wilcoxon signed-rank test, reporting the Hodges–Lehmann estimator (95% CI). Treatment N showed a significant increase (H–L Δ = 6.0 mm, 95% CI 2.5–9.5 mm; Holm-adjusted *p* < 0.05), and Treatment S also showed a significant increase (H–L Δ = 10.5 mm, 95% CI 6.5–14.5 mm; Holm-adjusted *p* < 0.05) (Fig. 4). These within-treatment results align with the mixed-effects analysis, indicating greater improvement under S than under N.

## Discussion

Consistent with our a priori working hypothesis that transcutaneous interferential current stimulation may influence masticatory performance, we observed immediate, within-session gains in occlusal function. Glucose elution concentration, which we employed as the primary endpoint, has been validated as a reliable indicator of masticatory and occlusal function in previous studies, showing significant correlations with conventional measures such as the sieve method^8^. In the primary endpoint, glucose elution concentration increased after IW stimulation (Pre 257 mg/dL → Post 288 mg/dL; mean difference + 31.5 mg/dL, p < 0.001), whereas it decreased under sham treatment (Pre 263 mg/dL → Post 243 mg/dL; mean difference − 19.8 mg/dL, p = 0.0019). The secondary endpoint (VAS) also improved within both treatments, with a larger median gain under IW stimulation (Hodges–Lehmann Δ 10.5 mm vs 6.0 mm), and mixed-effects modeling confirmed a superior effect of IW stimulation after adjusting for period and sequence. Taken together, these results indicate that IW stimulation acutely enhances masticatory performance, consistent with an immediate increase in occlusal force.

A plausible mechanism for the IW stimulation–related improvement in masticatory performance is afferent-driven facilitation of jaw-closer motoneurons. Human jaw-closing muscles contain abundant muscle spindles that provide finely graded proprioception; their discharge scales with contraction via α–γ co-activation, supplying excitatory drive to closer motoneurons during chewing^9,10^. Periodontal mechanoreceptors (PMRs) and muscle spindles provide convergent afferent input to trigeminal sensorimotor circuits and jointly calibrate bite force and jaw position on a cycle-by-cycle basis during mastication^9,11,12^. Importantly, peripheral sensory stimulation does not necessarily bias sensorimotor integration toward facilitation. The sign of PMR feedback is stimulus-rate–dependent: slowly rising inputs bias excitation of masseter units, whereas rapidly rising “tap-like” inputs evoke inhibition^9,11,13,14^. Depending on baseline excitability, task demands, and stimulation parameters, sensory input may enhance, diminish, or have no measurable effect on motor output. Therefore, the observed improvement in masticatory performance in the present study should be regarded as an empirical finding rather than an a priori predicted outcome. Different stimulation intensities, frequencies, or electrode configurations could plausibly yield neutral or even inhibitory effects on mastication, an issue that warrants systematic investigation in future studies.

Previous randomized, sham-controlled studies have demonstrated that transcutaneous electrical stimulation applied to the masticatory region can acutely influence masticatory muscle performance compared with sham stimulation^15,16^. When delivered near the perceptual threshold, interferential-current sensory stimulation would be expected to preferentially recruit low-threshold cutaneous/periodontal afferents and bias trigeminal reflex pathways toward excitation, thereby elevating occlusal output and stabilizing chew-cycle timing. Previous animal studies have demonstrated neuromodulatory effects of interferential current stimulation on brainstem swallowing circuits. In decerebrate guinea pigs, interferential current stimulation facilitated swallowing by enhancing sensory afferent transmission to the nucleus tractus solitarius and activating swallowing-related neurons within the central pattern generator^17^. However, the present human study did not directly assess neural activity, and thus mechanistic interpretations remain speculative.

Most previous studies examining the effects of electrical stimulation on the masticatory system have applied neuromuscular electrical stimulation (NMES) at intensities sufficient to elicit visible muscle contractions. For example, NMES of the masseter and temporalis muscles has been shown to increase EMG activity in stroke patients^18^, to enhance bite force and muscle thickness in older adults^19^, and to relieve pain or improve maximal occlusal force in patients with temporomandibular disorders^16^. While these findings provide important evidence that peripheral electrical stimulation can modulate oral motor function, they primarily rely on supra-threshold stimulation that directly activates motor units. By contrast, the present study, in line with the work of Iizumi and colleagues^5^, deliberately applied interferential current stimulation at intensities near the perceptual threshold or just below each participant’s discomfort threshold. This methodological distinction is crucial: rather than inducing overt muscle contractions, the stimulation was designed to engage sensory afferents without eliciting visible muscle activation. Such an approach minimizes discomfort, reduces the likelihood of fatigue or pain-related confounders, and is more suitable for translational use in routine dental or geriatric settings.

Interferential stimulation to the neck set just below each participant’s discomfort threshold has been associated with improvements in both mastication and swallowing^5^. In our study, the practitioner noted that immediately after stimulation participants perceived smoother chewing, and operative observation suggested a faster “tuning” (jaw opening–closing) cycle with a resultant increase in chewing counts. This pattern is compatible with a stimulation-related increase in the velocity of chew (VOC)^5^, which could elevate glucose extraction by accelerating comminution and enhancing saliva–bolus mixing. Because glucose elution was assessed immediately after the stimulation period, these kinematic changes likely persisted into the measurement window and may partly account for the observed increase, alongside other physiological influences. Future work should instrument jaw kinematics (e.g., VOC, cycle time) to quantify these contributions.

In addition, interferential current stimulation may influence local blood flow or autonomic activity, which could indirectly affect muscle performance or salivary secretion. Interferential current applied transcutaneously to the submandibular region has been shown to increase saliva production in patients with xerostomia (≈130% vs. no stimulation), but not in healthy subjects^20^, indicating that such stimulation can augment salivation under certain physiological conditions. Although our electrode placement targeted the masseter region rather than the salivary glands, current spread and/or autonomic reflexes could still have promoted salivary secretion during or immediately after stimulation. Because the gummy-jelly glucose extraction method is modestly sensitive to saliva volume, with salivary secretion rising over 10–20 s of chewing and inflating measured glucose extraction by roughly 3–5% in that window^21^, any IW-related increase in salivation may have slightly elevated apparent masticatory performance even if true comminution was unchanged. While such mechanisms are unlikely to fully account for the magnitude and consistency of the treatment effect, future studies should incorporate salivary flow measurements or saliva-normalized analyses.

Several limitations warrant consideration. First, the present study employed a sham-off control rather than an active control condition, and therefore nonspecific sensory cues or expectancy effects related to device activation cannot be fully excluded. Second, although investigators did not provide feedback to participants, the presence of a clinician observing chewing behavior may have introduced unintentional cues. Future studies should incorporate active control stimulation at non-masticatory sites and automated measurements such as jaw kinematics, bite force, or electromyography to strengthen causal inference. Third, randomization was implemented locally with sealed envelopes rather than centralized or computer-generated allocation. While the crossover design (each participant serving as his/her own control) and model adjustment for period and sequence mitigate between-participant imbalances and order effects, envelope-based procedures can be more susceptible to selection bias if concealment is compromised.

In conclusion, this randomized crossover study shows that IW stimulation during DFT produces immediate, within-session gains in masticatory performance, with concordant improvements in the objective (glucose elution) and subjective (VAS) endpoints and superiority to sham after adjustment for period and sequence. The pattern of effects is consistent with a physiological influence of peripheral electrical stimulation on masticatory performance, rather than being fully explained by expectancy effects or simple warm-up. Ancillary pathways such as stimulation-related acceleration of chewing and, to a lesser extent, changes in salivary flow may have contributed, but these are unlikely to fully account for the magnitude and consistency of the observed effects. Future work should quantify jaw kinematics and direct force/muscle readouts (e.g., VOC, bite-force dynamometry, EMG) and incorporate salivary flow measures or saliva-normalized analyses, while testing dose–response, electrode placement, and durability/generalizability across sessions. If replicated and durable, IW stimulation could represent a rapid, noninvasive adjunct to conventional oral rehabilitation in populations at risk of dysphagia.

## Methods

### Ethics approval and consent to participate

The protocol was approved by the Ethics Committee of Hyogo Medical University (approval No. 5051; 2025-06-12), and all procedures complied with relevant guidelines/regulations and the Declaration of Helsinki. Written informed consent was obtained from all participants.

### Study design

This study was designed as a randomized crossover trial. Participants were recruited from patients who attended regular dental checkups at Hara Dental Clinic. The intervention involved transcutaneous IW stimulation using a commercially available facial device (Cellcure DFT, JCraft Inc., Japan). IW stimulation consisted of two medium-frequency sinusoidal carriers (3.6–4.0 kHz) applied to produce a 400 Hz interferential (beat) frequency, delivered in intermittent trains with a 1,300 ms ON and 200 ms OFF cycle (1.5 s period; ~ 86.7% duty cycle). De-identified data were transferred with password protection to Y. O. for analysis.

This trial was prospectively registered in the University Hospital Medical Information Network Clinical Trials Registry (UMIN-CTR; ID: UMIN000058249, registered on 1 July 2025; direct link: https://center6.umin.ac.jp/cgi-open-bin/ctr/ctr_view.cgi?recptno=R000066586).

### Participants

Eligible participants were adults aged 30–90 years attending routine dental examinations who met the following inclusion criteria:

- No underlying systemic diseases affecting masticatory function.
- Not using dentures, or only using prosthetic devices with equivalent function to natural teeth.
- No prior IW stimulation within the past three months.
- Ability to understand the study purpose and provide written informed consent.

Exclusion criteria included regular use of home facial devices, temporomandibular disorders, xerostomia, metal allergies, dermatological contraindications to electrical stimulation, pregnancy or lactation, ongoing major dental treatment, pacemaker use, cardiovascular or bleeding disorders, peripheral neuropathy, facial neuralgia, or other conditions deemed inappropriate by the investigators.

Participants were recruited between 21 June 2025 and 19 August 2025. Each intervention phase was followed only by immediate outcome assessment, and no long-term follow-up was conducted.

### Randomization and allocation concealment

For the two-period crossover (stimulation (**S**) followed by sham (**N**), or **N** followed by **S**), assignment was implemented onsite using sequentially numbered, opaque, sealed envelopes (SNOSE). The treating dentist (enrolling clinician) instructed procurement of the envelopes and A/B assignment cards, after which clinic administrative staff prepared the envelopes by inserting preprinted “A” (S → N) or “B” (N → S) cards according to one of three pre-specified randomization schedules kept on file at the clinic; the particular schedule used to prepare the envelopes was not disclosed to the investigators. Envelopes were stored securely and opened in order after baseline assessments by the treating dentist. Although allocation was not centrally administered or computer-generated, this procedure preserved allocation concealment at the point of assignment. Period and sequence were prespecified fixed effects in the primary model.

### Masking

Participants were masked to assignment; the device display/handling were identical across conditions, and no cues were provided. Approximately 20% of participants reported no perceptible sensation even at level 6 stimulation, about 30% reported only slight sensation, and the remaining 50% clearly perceived stimulation. Outcome assessors were not blinded.

### Washout period

Each intervention phase was separated by one month (± 5 days). Acute carry-over effects of transcutaneous electrical stimulation on the masticatory system are short-lived: most trials assess outcomes immediately after a single 20–60-min session^15,16^, and when quantified, post-stimulation analgesia typically persists only for a few hours (≈3–4 h)^22^. Although some temporomandibular disorder studies report short-term changes detectable up to 24–48 h after a single longer session^23^, durable adaptations generally require multi-week training protocols rather than a single exposure. In addition, salivation-related effects are stimulus dependent and transient: chewing evokes rapid increases in salivary flow that peak within the first minute and decay over subsequent minutes, with no sustained elevation once stimulation ceases^24–26^. Accordingly, a one-month washout far exceeds the expected durations of any acute stimulation-related effects, including transient changes in masticatory performance or salivary responses, and minimizes any carry-over between periods.

### Study procedure

At each visit, baseline data (age, sex, and dental history) were recorded. Masticatory performance was assessed using a standardized gummy jelly (8% gelatin, 2 g)^8^. Participants chewed the jelly for 20 s, rinsed with 10 mL distilled water, and expectorated the mixture into a filter-equipped cup. The amount of glucose eluted was measured with a glucose sensor (Glucosensor GS-1, GC Corp., Japan).

- Stimulation (**S**) group: IW stimulation was applied for 2.5 min per side (total 5 min) with the device head placed on the buccal masseter region (from the zygomatic arch to the mandibular angle). The stimulation was delivered at level 6 (2.98–5.96 mA). This level was selected because stimulation above 6.5 often caused strong discomfort requiring intensity reduction, whereas many participants did not perceive the stimulation at or below level 5.5.
- Sham, no stimulation (**N**) group: The device was applied in the same manner without current flow.

Five minutes after the intervention, participants repeated the gummy jelly test, and glucose elution was measured again. At the subsequent visit, participants underwent the opposite intervention following the same protocol.

### Outcomes

The primary outcome was glucose concentration (mg/dL) released from the gummy jelly, as measured by the glucose sensor. This measure has been validated as an effective indicator of occlusal and masticatory function. Previous work demonstrated that the amount of glucose extraction from gummy jelly chewing is significantly and positively correlated with conventional masticatory performance values obtained by the sieve method^8^, confirming its utility as an objective and clinically applicable assessment of masticatory ability.

The secondary outcome was subjective evaluation of masticatory performance, assessed using a visual analogue scale (VAS). Participants were asked to place a mark on a 100-mm horizontal line, where 0 mm represented “cannot chew at all” and 100 mm represented “extremely easy to chew.” The distance from the 0 mm anchor to the participant’s mark was then measured in millimeters and used as the VAS score.

### Statistical analysis

The primary analysis was conducted using a linear mixed-effects crossover model:

$$\Delta \sim \text{Treatment }+\text{ Period }+\text{ Sequence }+ (1|\mathrm{ID})$$

where $\Delta$ represents the change in glucose concentration (post–pre within each session). Treatment (stimulation vs. sham), period, and sequence were modeled as fixed effects, with subject ID as a random intercept. This model was implemented in R (version 4.5.1, lme4 package). Degrees of freedom and p-values were computed using the Satterthwaite approximation via lmerTest v3.1.3 in R 4.5.1 with lme4 v1.1.37.

For sub-analyses, the following methods were applied:

- For the primary outcome (glucose elution), within-treatment pre–post changes were evaluated by paired t-tests for stimulation and sham conditions.
- For the secondary outcome (VAS scores), normality was assessed using the Shapiro–Wilk test. If the distribution deviated from normality, the Wilcoxon signed-rank test was applied to evaluate within-treatment pre-post changes.
- Within-treatment pre–post tests were considered descriptive; two comparisons were additionally examined with Holm adjustment.

All tests were two-tailed, and a *p* value < 0.05 was considered statistically significant.

### Sample size rationale

Feasibility supported a target of ~ 40–50 participants for the two-period crossover. A priori, we specified a minimal clinically important difference (MCID) of 30 mg/dL for the within-subject change in glucose elution and assumed a conservative SD of 60 mg/dL. Under a two-sided α = 0.05 paired design, this corresponds to 80% power with n≈32. Our actual n = 46 therefore affords ≈92% power to detect the MCID and an expected 95% confidence-interval half-width of ~ 17–18 mg/dL, meeting a pragmatic precision target (≤ 20 mg/dL).

### Use of AI assistance

Portions of the manuscript text were drafted and edited with assistance from ChatGPT (OpenAI; accessed on 24 Aug 2025) based on author-provided outlines, study protocols, and statistical outputs. All authors critically reviewed, revised, and approved the final text, and take full responsibility for the content. No AI tools were used to generate, analyze, or alter the data, statistical results, figures, or references.

## Data Availability

De-identified individual-level data, the data dictionary, and figure source data are available at Zenodo (DOI:10.5281/zenodo.18176543).

## Code availability

The analysis code used in this study is archived at the same Zenodo record (10.5281/zenodo.18176543).
